# Characterization of real‐world treatment practices and outcomes among patients with chronic lymphocytic leukemia treated in a Finnish tertiary center

**DOI:** 10.1002/jha2.322

**Published:** 2021-11-21

**Authors:** Juha Ranti, Katariina Perkonoja, Tommi Kauko, Heidi Loponen, Emmi I. Joensuu, Tiina M. Järvinen

**Affiliations:** ^1^ Department of Hematology and Stem Cell Transplantation Unit Division of Medicine Turku University Hospital Turku Finland; ^2^ Auria Clinical Informatics Turku University Hospital, Hospital District of Southwest Finland Turku Finland; ^3^ MedEngine Oy Helsinki Finland; ^4^ Medical Affairs Janssen‐Cilag Oy Espoo Finland

**Keywords:** chemoimmunotherapy, chronic lymphocytic leukemia, IGHV, survival, targeted therapy

## Abstract

**Objectives:**

We conducted this retrospective study to characterize the change in chronic lymphocytic leukemia (CLL) treatment patterns between 2005 and 2019, to understand the treatment sequencing across the course of the disease, and to investigate how targeted agents and prognostic testing were implemented into the patient care.

**Methods:**

This study included adult patients with CLL treated at the Hospital District of Southwest Finland during the study period. Data were collected from the Turku University Hospital data lake.

**Results:**

In total, 122 and 60 patients received first‐ and second‐line treatments for CLL, respectively. The shift from conventional chemoimmunotherapy to targeted treatments in recent years (2014–2019) was observed. The median overall survival times were not reached in patients treated with targeted agents compared to conventional standard treatments in first‐ and second‐line settings and improved toward the end of the study period. Prognostic testing increased during the study follow‐up and patients with unmutated immunoglobulin heavy‐chain variable showed significantly poorer overall survival and time‐to‐next‐treatment outcomes than patients with mutated immunoglobulin heavy‐chain variable.

**Conclusions:**

This real‐world study implicated added value of targeted chemo‐free therapies as reported in randomized clinical trials, and highlighted the necessity of prognostic testing in order to improve treatment selection and patient outcomes.

## 1 INTRODUCTION

Chronic lymphocytic leukemia (CLL) is the most common adult leukemia in western countries. In Finland, 2900 individuals live with CLL currently and the age‐adjusted incidence was 5.09 per 100,000 people in 2019 [1]. CLL is a malignancy of B‐cells characterized by CD5‐positive lymphocytes accumulating in the blood and lymphoid organs [2, 3]. Various chromosomal alterations have proven to be prognostically important and can guide treatment selection [2, 4].

Since early 2010, the treatment armamentarium has been expanded by the addition of targeted therapies besides conventional chemoimmunotherapy (CIT) regimens (e.g., fludarabine‐cyclophosphamide‐rituximab [FCR] and bendamustine‐rituximab [BR]) [5, 6, 7, 8]. These targeted therapies, such as the BTK‐inhibitor ibrutinib, PI3K‐inhibitor idelalisib, and BCL2‐inhibitor venetoclax, have diminished the role of conventional CIT in CLL treatment [9], and the improved outcomes with targeted agents have been described both in untreated and relapsed settings [10, 11, 12, 13, 14, 15, 16, 17, 18].

The most common prognostic markers in CLL are chromosomal alterations such as del(13q), del(11q), del(17p), trisomy 12, and TP53 mutation, as well as the mutation status of immunoglobulin heavy‐chain variable (IGHV) genes. Traditionally, high‐risk CLL is defined as having del(11q), unmutated IGHV gene, and/or TP53 aberration (TP53 mutation or del[17p]) [19]. Especially the patients harboring TP53 aberration are relatively refractory to conventional CIT [4, 20]. As a consequence, CIT is currently the most beneficial in the first‐line treatment of young fit patients with mutated IGHV and without a TP53 aberration [9, 21]. As the effectiveness of targeted therapies is not substantially dependent on conventional prognostic variables (e.g., disease state, age, and general fitness), the characterization of the disease's molecular biology has become increasingly important. According to the International Workshop on Chronic Lymphocytic Leukemia (iwCLL) and European Society for Medical Oncology guidelines, assessment of cytogenetic abnormalities and testing for TP53 mutations and IGHV status should be applied in routine clinical practice to guide the treatment selection alongside the patient's clinical stage and symptoms [4, 21]. Over time, Finnish treatment guidelines have aligned with international recommendations [4, 8]. In addition, Finnish treatment practices reflect the national reimbursement decisions. In recent years, the reimbursement of ibrutinib, idelalisib, and venetoclax in relapsed CLL and in CLL with TP53 aberration has translated into growing numbers of patients receiving targeted therapy.

Amid the changing tides of CLL therapy, up‐to‐date data on routine clinical care in Finland was lacking, while these data from other Nordic countries are available [22, 23, 24]. Therefore, we conducted this retrospective cohort study to characterize evolving CLL treatment patterns in one Finnish tertiary center. The primary aims of this study were to describe CLL treatment practices in the Hospital District of Southwest Finland between 2005 and 2019, understand treatment sequencing across the course of the disease, and investigate how targeted agents are utilized in disease management after their reimbursement and introduction into the Finnish healthcare system from 2015 onward. As secondary outcomes, overall survival (OS) and time‐to‐next‐treatment (TTNT) were assessed to evaluate the effectiveness of targeted treatments compared to historically used conventional therapies. Additionally, the utilization of prognostic testing was evaluated.

## 2 MATERIALS AND METHODS

### 2.1 Study population and data collection

This retrospective, observational cohort study of Finnish CLL patients was designed to characterize treatment practices, treatment outcomes, and the utilization and impact of prognostic testing in real‐life clinical practice. This study included adult patients with CLL (ICD‐10: C91.1, ≥18 years of age) who received at least one treatment at the Hospital District of Southwest Finland (population base 470,000) between January 1, 2005 and June 30, 2019. Patients were excluded if treatment or treatment line could not be verified, or if the patient had received a stem cell transplant (autologous or allogeneic). Patients were followed from the initiation of the first‐line treatment until the end of the study period (June 30, 2019) or death, whichever came first. Data were collected retrospectively from the Turku University Hospital data lake.

The data collected at baseline and/or during follow‐up included: age, sex, date of CLL diagnosis, Binet stage, comorbidities (ICD‐10), cytogenetic lesions (del[11q], trisomy 12, del[13q], and del[17p]), mutation status (TP53 and IGHV), name of received therapy, and date of death.

### 2.2 Outcome measures

Treatment regimens were categorized as follows: fludarabine‐cyclophosphamide or FCR (FC/FCR), bendamustine or BR (B/BR), chlorambucil‐based therapy (monotherapy or in combination with obinutuzumab, ofatumumab, or rituximab), monoclonal antibody‐based therapy (monotherapy or in combination other than FCR or BR), targeted therapy (ibrutinib, idelalisib, or venetoclax as monotherapy or in combination), or other therapy excluding regimens in any other category.

OS was defined as the length of survival in months from the initiation of first‐ or second‐line treatment until the date of death, and the patients alive at the end of follow‐up were right‐censored. TTNT was defined as the time in months from one treatment regimen initiation to the initiation of the next treatment, and the patients without the next treatment were right‐censored.

In the subgroup analyses patients were stratified based on the year of their first‐ or second‐line treatment initiation to early (between 2005 and 2013) or late (between 2014 and 2019) periods. In addition, patients were categorized as targeted treatment or historical standard of care (SOC) groups, based on the treatment received. The patients in the targeted group had received ibrutinib, idelalisib, or venetoclax, and patients in the SOC group had received any other treatment regimens.

Patients were additionally categorized based on IGHV mutational status (mutated and unmutated) and del(17p) and/or TP53 mutation (with TP53 aberration and without TP53 aberration), or unknown if the status was not tested. Patients with 17p or 11q deletion, TP53 mutation, or unmutated IGHV were categorized as high‐risk and patients without as low‐risk.

### 2.3 Statistical analyses

The descriptive findings for continuous variables were reported as medians along with lower and upper quartiles (Q1–Q3), except for age for which range (min–max) was used instead of quartiles, and categorical variables were reported as observed proportions and frequencies. Treatment regimens at first‐, second‐, and third‐line were stratified by the treatment initiation period and/or treatment regimen. The follow‐up times, including all patients, not just event‐free, were calculated for each subgroup from the beginning of each treatment line to the end of the follow‐up (Table S1).

The OS and TTNT were assessed from the initiation of first‐ or second‐line treatments and explored using the Kaplan‐Meier estimator. The median OS (mOS) and median TTNT (mTTNT) with 95% confidence intervals were reported if reached. These values indicate the time point by which half of the patients in the group are still alive or have initiated the next treatment, respectively. The differences in survival distributions were compared using the log‐rank test. OS‐ and TTNT‐specific hazard ratios (HRs) with 95% confidence intervals (CIs) were estimated using multivariate weighted Cox proportional hazard regression models adjusted for patient characteristics (presented in Table 1) at the start of the first treatment line and the treatment regimen given within the treatment line (historical SOC therapy vs. targeted therapy groups) [25]. Missing data were described and used without imputation in the analyses. The statistical significance (*P*) threshold was set at 0.05. All analyses were conducted with R software version 3.6.3 [26].

**TABLE 1:** Patient characteristics at the initiation of first‐ and second‐line treatments during different time periods (2005–2013, 2014–2019, and 2005–2019)

	First‐line treatment initiation			Second‐line treatment initiation		
Patient characteristics	**Early period 2005–2013 (*N* = 74)**	**Late period 2014–2019 (*N* = 48)**	**Total 2005–2019 (*N* = 122)**	**Early period 2005–2013 (*N* = 27)**	**Late period 2014–2019 (*N* = 33)**	**Total 2005–2019 (*N* = 60)**
Age (y)						
Median (range)	70.5 (44.0–89.0	68.5 (36.0–86.0)	69.0 (36.0–89.0)	68.0 (51.0–87.0)	68.0 (49.0–82.0)	68.0 (49.0–87.0)
18–64, *n* (%)	27 (36.5)	17 (35.4)	44 (36.1)	12 (44.4)	11 (33.3)	23 (38.3)
65–74, *n* (%)	19 (25.7)	21 (43.8)	40 (32.8)	7 (25.9)	15 (45.5)	22 (36.7)
≥75, *n* (%)	28 (37.8)	10 (20.8)	38 (31.1)	8 (29.6)	7 (21.2)	15 (25.0)
Gender, male, *n* (%)	50 (67.6)	39 (81.2)	89 (73.0)	18 (66.7)	27 (81.8)	45 (75.0)
Binet stage C						
*n* (%)	32 (43.2)	16 (33.3)	48 (39.3)	13 (48.1)	12 (36.4)	25 (41.7)
Missing, *n* (%)	0 (0)	1 (2.1)	1 (0.8)	0 (0)	0 (0)	0 (0)
Comorbidity index^a^						
0, n (%)	15 (20.3)	16 (33.3)	31 (25.4)	8 (29.6)	10 (30.3)	18 (30.0)
1–2, *n* (%)	40 (54.1)	16 (33.3)	56 (45.9)	12 (44.4)	15 (45.5)	27 (45.0)
≥3, *n* (%)	19 (25.7)	16 (33.3)	35 (28.7)	7 (25.9)	8 (24.2)	15 (25.0)
Cytogenetic lesions (FISH)						
Del(11q)						
Positive, *n* (%)	9 (12.2)	7 (14.6)	16 (13.1)	4 (14.8)	7 (21.2)	11 (18.3)
Unknown, *n* (%)	37 (50.0)	11 (22.9)	48 (39.3)	13 (48.1)	4 (12.1)	17 (28.3)
Trisomy 12						
Positive, *n* (%)	5 (6.8)	4 (8.3)	9 (7.4)	3 (11.1)	5 (15.2)	8 (13.3)
Unknown, *n* (%)	37 (50.0)	11 (22.9)	48 (39.3)	13 (48.1)	4 (12.1)	17 (28.3)
Del(13q)						
Positive, *n* (%)	17 (23.0)	18 (37.5)	35 (28.7)	7 (25.9)	14 (42.4)	21 (35.0)
Unknown, *n* (%)	37 (50.0)	11 (22.9)	48 (39.3)	13 (48.1)	4 (12.1)	17 (28.3)
Del(17p)						
Positive, *n* (%)	6 (8.1)	4 (8.3)	10 (8.2)	4 (14.8)	4 (12.1)	8 (13.3)
Unknown, *n* (%)	33 (44.6)	2 (4.2)	35 (28.7)	11 (40.7)	2 (6.1)	13 (21.7)
TP53 mutation						
Mutated, *n* (%)	2 (2.7)	6 (12.5)	8 (6.6)	0 (0)	6 (18.2)	6 (10.0)
Unmutated, *n* (%)	7 (9.5)	28 (58.3)	35 (28.7)	1 (3.7)	18 (54.4)	19 (31.7)
Unknown, *n* (%)	65 (87.8)	14 (29.2)	79 (64.8)	26 (96.3)	9 (27.3)	35 (58.3)
IGHV mutational status						
Mutated, *n* (%)	10 (13.5)	15 (31.2)	25 (20.5)	4 (14.8)	7 (21.2)	11 (18.3)
Unmutated, *n* (%)	27 (36.5)	22 (45.8)	49 (40.2)	12 (44.4)	23 (69.7)	35 (58.3)
Unknown, *n* (%)	37 (50.0)	11 (22.9)	48 (39.3)	11 (40.7)	3 (9.1)	14 (23.3)

FISH, fluorescence in situ hybridization; IGHV, immunoglobulin heavy chain variable gene; missing, status not known; TP53, tumor protein 53 gene; unknown, cytogenetic lesion, TP53 mutation or IGHV mutational status not tested.

^a^ The comorbidity index was defined according to the Charlson comorbidity index [37] with the range of 0–10 in the dataset.

### 2.4 Ethical considerations

The study approval was obtained from the Hospital District of Southwest Finland (T288/2019) and was performed in accordance with the declaration of Helsinki and in compliance with applicable national laws. According to Finnish legislation, informed consent is not required for studies based on patient records.

## 3 RESULTS

### 3.1 Patient characteristics

Altogether, 122 patients initiated first‐line treatment during the study period (Table 1). The median age at first‐line treatment initiation was 69 years (range 36–89). At first‐line initiation between 2005 and 2019, cytogenetic lesions del(11q), trisomy 12, del(13q) and del(17p) were present in 21.6% (16/74), 12.2% (9/74), 47.3% (35/74), and 11.5% (10/35) of the tested individuals, respectively. TP53 mutation was detected in 18.6% and unmutated IGHV in 66.2% of the tested patients. The testing for cytogenetic lesions or mutations was not routinely performed during the study period.

Second‐line treatment was initiated for 60 patients in the total cohort with a median age of 68 years (range 49–87) at the initiation (Table 1). Cytogenetic lesions del(11q), trisomy 12, del(13q), and del(17p) were present in 25.6% (11/43), 18.6% (8/43), 48.8% (21/43), and 17.0% (8/47) of the tested patients, respectively. TP53 mutation was detected in 24.0% and unmutated IGHV in 76.1% of the tested second‐line therapy patients.

### 3.2 Treatment pattern changes over time

**FIGURE 1:**
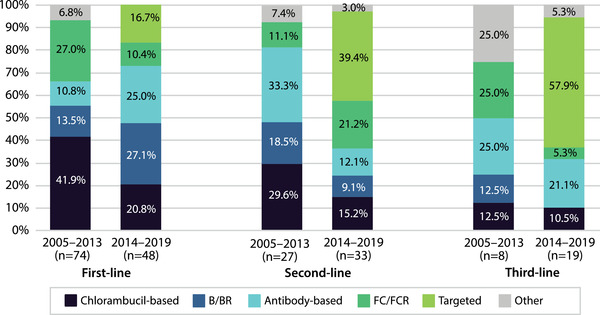
First‐, second‐, and third‐line treatments during 2005–2013 and 2014–2019. Percentages of treatment regimens used during 2005–2013 and 2014–2019

In the early period (during 2005–2013), chlorambucil‐based therapy was the most frequently used first‐line treatment (41.9% [31/74]) followed by FC/FCR (27.0% [20/74]) (Figure 1). The use of both chlorambucil‐based regimens (20.8% [10/48]) and FC/FCR (10.4% [5/48]) decreased during the late period (2014–2019), while the use of B/BR (27.1% [13/48] in the late vs. 13.5% [10/74] in the early period) and antibody‐based therapies (25.0% [12/48] vs. 10.8% [8/74]) increased. In the late period, 16.7% (8/48) of patients used targeted therapies as first‐line treatment.

In the early period, the most common second‐line treatments were antibody‐based (33.3% [9/27]) or chlorambucil‐based regimens (29.6% [8/27]), and B/BR (18.5% [5/27]). During the late period the use of chlorambucil‐based therapy decreased (15.2% [5/33]), while the most frequently used regimens were targeted therapies (39.4% [13/33]) and FC/FCR (21.2% [7/33]) (Figure 1).

A third treatment line was initiated for eight patients in the early period and for 19 patients in the late period. Antibody‐ and fludarabine‐based therapies were the most frequently used third‐line regimens in the early period (both 25.0% [2/8]). In the late period, targeted therapies were used by more than half (57.9% [11/19]) and antibody‐based therapies by 21.1% (4/19) of the patients (Figure 1).

**FIGURE 2:**
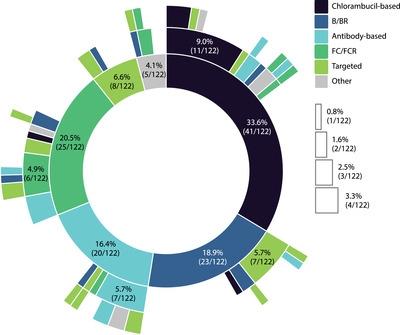
Treatment sequencing during the study period (2005–2019). Sunburst plot illustrating treatment sequencing across treatment lines. The innermost circle represents the first‐line treatment. The white boxes indicate the number of patients (*n* < 5) and percentages for the respective colored sectors not labeled in the plot

An overview of treatment sequencing throughout the study period (2005–2019) is shown in Figure 2 and Table S2.

### 3.3 First‐line treatment outcomes

**FIGURE 3:**
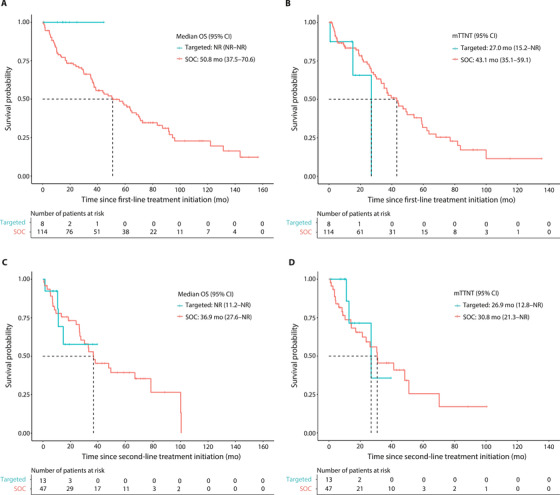
Kaplan‐Meier survival curves for overall survival (OS) and time‐to‐next‐treatment (TTNT) for targeted or standard of care therapies in the first‐ and second‐line treatments. (A) OS and (B) TTNT in first‐line treatment (C) OS and (D) TTNT in second‐line treatment. Targeted therapies: ibrutinib, idelalisib, or venetoclax (monotherapy or in combination); standard of care (SOC) therapies: any other therapy. Statistical comparisons were not conducted due to the small sample size in the targeted group

In the first treatment line, the follow‐up was limited (median follow‐up time 16.0 months) for patients treated with targeted therapies (*n* = 8; majority on venetoclax and a few patients on idelalisib or ibrutinib) and all patients were alive at the end of the follow‐up, whereas 63.2% of the patients treated with historical SOC therapies (*n* = 114) were deceased (median follow‐up 36.8 months). The mOS was not reached in the targeted group and was 50.8 months (95% CI, 37.5–70.6) in the SOC group (Figure 3A). During the follow‐up, 37.5% of the patients in the targeted group and 50.0% in the SOC group received second‐line treatment (Table S2). The mTTNT was 27.0 months (95% CI, 15.2–not reached [NR]) in the targeted group and 43.1 months (95% CI, 35.1–59.1) in the SOC group (Figure 3B). These results are based on a small number of patients in the targeted group and due to the sample size, statistical comparisons could not be performed.

**FIGURE 4:**
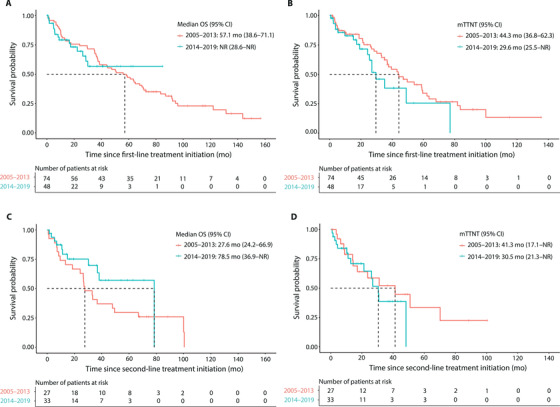
Kaplan‐Meier survival curves for overall survival (OS) and time‐to‐next‐treatment (TTNT) in first and second‐line treatments during 2005–2013 and 2014–2019. (A) OS (log‐rank *p*‐value = 0.872) and (B) TTNT (log‐rank *p*‐value = 0.106) in first‐line treatment, (C) OS (log‐rank *p*‐value = 0.151) and (D) TTNT (log‐rank *p*‐value = 0.459) in second‐line treatment

The mOS was 57.1 months (95% CI, 38.6–71.1) for patients whose treatment started in the early period, and was not reached in the late period during the follow‐up (Figure 4A). Second‐line treatment was initiated for 56.8% and 37.5% of the early and late period patients, respectively. The mTTNT was 44.3 months (95% CI, 36.8–62.3) and 29.6 months (95% CI, 25.5–NR) for patients whose treatment started during the early and late period, respectively (HR 1.62 [95% CI, 0.90–2.90], *p *= 0.108) (Figure 4B). Factors associated with OS and TTNT were also explored in the multivariate Cox model as presented in Supplementary Table 3.

### 3.4 Second‐line treatment outcomes

In the second treatment line, 69.2% of the patients in the targeted group (*n* = 13; mostly ibrutinib or venetoclax, and idelalisib for a few patients) were alive at the end of follow‐up (median follow‐up 11.2 months) and the mOS was not reached (Figure 3C). In the historical SOC group (*n* = 47), 38.3% of the patients were alive at the end of the follow‐up (median follow‐up 30.2 months) and the mOS was 36.9 months (95% CI, 27.6–NR) (Figure 3C). Out of the patients in the SOC group, 51.1% received subsequent treatment lines during the follow‐up, and the mTTNT was 30.8 months (95% Cl, 21.3–NR; Figure 3D). In contrast, only 23.1% of patients in the targeted group initiated third‐line treatment with the mTTNT of 26.9 months (95% CI, 12.8–NR; Figure 3D). Due to the small sample size in the targeted group, no statistical comparisons were carried out. In the multivariate analyses, targeted treatments significantly decreased the risk for subsequent treatment (HR 0.21 [95% CI 0.07–0.62], *p *= 0.005) (Table S3).

The mOS was 27.6 months (95% CI, 24.2–66.9) for patients whose second‐line treatment was initiated during the early period and 78.5 months (95% CI, 36.9–NR) for patients in the late period (Figure 4C). A third‐line treatment was initiated for 48.1% and 42.4% of the early and late period patients, respectively. The mTTNT was 41.3 months (95% CI, 17.1–NR) for patients treated in the early period and 30.5 months (95% CI, 21.3–NR) for patients in the late period (Figure 4D).

### 3.5 Treatment outcomes based on high‐risk disease features

In this study cohort, mOS and mTTNT were shorter in patients with unmutated IGHV in both first‐ and second‐line treatment without treatment stratification compared to patients with mutated IGHV (Table S4) reaching statistical significance in the first‐line treatment group (mOS, *p *= 0.005; mTTNT, *p *= 0.001). Multivariate analyses showed that unmutated IGHV significantly increased the risk for subsequent treatment (first‐line: HR 3.43 [95% CI, 1.46–8.02], *p *= 0.005; second‐line: HR 4.70 [95% CI, 1.16–19.01], *p *= 0.030) (Table S3).

In high‐risk patients (17p or 11q deletion, TP53 mutation, or unmutated IGHV) and in patients with TP53 aberration (TP53 mutation or 17p deletion), the mOS in first‐ and second‐line treatment was shorter compared to low‐risk patients or patients without TP53 aberration (Table S4). Of note, a high proportion of deaths was observed among patients with unknown prognostic test results (Table S4), which might indicate a significant number of patients in fact having high‐risk disease features in this group.

## 4 DISCUSSION

CLL treatment has changed over the last decades reflecting the advances in the treatment armamentarium. At first, the addition of CD20‐antibody to conventional chemotherapy improved patient outcomes, while more extensive benefits have been seen recently with novel targeted treatments becoming available [27, 28]. In this study, we set out to characterize CLL treatment practices, outcomes, and the role of prognostic testing in real‐life clinical practice from a well‐defined geographical region in Southwest Finland between 2005 and 2019.

In general, the patients in the present study had been treated in accordance with the Finnish and European treatment guidelines prevailing during the study period [8, 29]. Chlorambucil‐based therapy was widely used in the first‐line during the study period, which was in line with similar reports from Sweden [22]. On the other hand, the first‐line use of FC/FCR was lower than reported in another Finnish study [30]. The observed changes in the first‐line treatment patterns from 2005 to 2019 were consistent with the results reported from Finland and Sweden, which have shown the increasing use of B/BR and decreasing use of chlorambucil‐based therapy [22, 23, 30]. The implementation of targeted therapies during 2014–2019 was expected and reflects the reimbursement of targeted agents in Finland during this period.

The most used second‐line treatments during the early period were antibody‐based therapies (rituximab‐based combinations other than FCR or BR; e.g., R‐CHOP), chlorambucil‐based therapies, and B/BR. The second‐line use of chlorambucil‐based therapies decreased from early to late period, which corresponds to other reports from Nordic countries [23, 30]. The overall use of FC/FCR declined during the study but was seemingly still at a high percentage in second‐line treatment during the late period. However, the number of patients in the different treatment groups was small, and hence no conclusions can be drawn. During the late period, targeted treatments were the most used regimens in the second and third lines.

The superior efficacy of targeted treatments over conventional CITs has been demonstrated in several randomized clinical trials in both untreated and relapsed diseases [9, 10, 12, 13, 15, 16, 17, 18]. Of note, only ibrutinib has been compared to most recommended CIT alternatives across all patient segments in previously untreated and relapsed CLL [10, 12, 13, 17, 18], and significant OS benefit has been demonstrated over FCR and chlorambucil monotherapy in the first‐line treatment [12, 18]. It has also been shown, that by using CIT the long‐term survival is achieved only for a subset of patients, and over 50% of patients are relapsing, progressing, or deceased within five years of receiving CIT [31, 32]. In this study, patients receiving any targeted treatment were grouped together and thus, no conclusions concerning individual treatment regimens can be made. The mOS was not reached for patients treated with targeted therapies in either the first or second line, and a greater proportion of patients was alive at the end of follow‐up compared to patients on other treatments. The mOS was also longer among patients whose treatment was initiated during the late period compared to the early period, in agreement with previously published data from Finland [30]. In Swedish retrospective studies, no significant improvement in the OS was observed between 2003 and 2013 [22, 23].

The mTTNT in this study was shorter in the targeted therapy group and in patients who initiated treatment during the late period, which may indicate that during recent years it has become easier to change the treatment in case of intolerance or suboptimal response as several targeted treatment options are now available. A number of treatment switches were related to the use of idelalisib, which has been associated with severe treatment‐emergent adverse events [33]. Additionally, the targeted treatments were presumably distributed to patients with difficult‐to‐treat or rapidly progressing diseases. Due to the low number of events and patients in the targeted therapy group, statistical comparisons for OS and TTNT could not be performed.

Unmutated IGHV is known to be a negative predictor of survival [24, 34]. Clinical trials show that progression‐free survival benefit is achieved in patients with unmutated IGHV using targeted treatments in the first‐line compared to conventional therapy [10, 12, 15, 17, 18]. IGHV testing was recommended by the iwCLL in 2018 and introduced shortly after that to the Finnish treatment guidelines in which the testing was recommended at the physician's discretion during the study period [4, 29]. At the Hospital District of Southwest Finland, testing for IGHV status has been available prior to 2018, but not in routine use for the entire study period, which was reflected by the fact that IGHV mutation status was unknown in 48% of patients in 2005–2013 and 17% of the patients in 2014–2019. The OS and TTNT results observed for patients whose IGHV status was known were concordant with published data, showing significantly poorer outcomes in the population with unmutated IGHV disease [31, 35]. Interestingly, first‐line mOS was shortest for the subset of patients whose IGHV status was unknown, suggesting that these patients may not have received optimal treatment. In order to provide patients treatment, they will benefit the most from, the IGHV mutation status should be investigated prior to the treatment initiation, as advised by the iwCLL and European Society for Medical Oncology guidelines [4, 21].

In addition to IGHV mutation status, molecular cytogenetics for del(13q), del(11q), del(17p), and trisomy 12, as well as TP53 mutation, should always be examined at the baseline [4]. Testing is paramount as targeted therapies confer consistent long‐term disease control and progression‐free survival benefit in patients with high‐risk CLL, where benefit with traditional chemotherapy or CIT is limited [4, 10, 11, 12, 15, 17, 18]. In this study, the testing for cytogenetic lesions and TP53 mutation status increased in general during the study follow‐up. However, these markers, especially TP53 that was not routinely tested until 2018, were unknown for a large subset of patients. Recent real‐world data from the informCLL registry from the USA show that low rates of prognostic testing may prevent patients from getting the most appropriate therapy, and even when prognostic testing was conducted, a large subset of high‐risk patients (with TP53 aberration) was treated with CIT that did not align with treatment recommendations [36]. These observations highlight the necessity to increase prognostic testing and the utilization of that knowledge to guide optimal treatment decisions.

This study provides novel information on CLL treatment practices in Finland and is the only observational study conducted in Finland in which the follow‐up period extends to the era of targeted therapies. Additionally, the current study is the most comprehensive Finnish IGHV and TP53 dataset available. The previous observational study describing CLL treatment patterns in Finland reported IGHV mutation status only for a minority of the study population and TP53 results were not reported [30]. Moreover, the results of this real‐world study are in line with other Nordic publications [22, 23].

This study was limited by a relatively small number of patients and short follow‐up times, especially in the targeted therapy group and during the late period. The follow‐up period for targeted therapies was short compared to other regimens since idelalisib in combination with rituximab was reimbursed in late 2015 and ibrutinib or venetoclax as monotherapy, not until 2018. Additionally, targeted therapies were reimbursed in the first‐line for patients with del(17p) or TP53 mutation only during the study period. Due to this inherent bias, and a high number of patients with unknown prognostic testing results, the OS comparisons may not be meaningful after a certain time point and the results describing the effect of different high‐risk disease features on the treatment outcomes are speculative only. The predominant usage of conventional treatments throughout the study period further hampers the assessment of true effectiveness between novel drugs and historical SOC.

This was a single‐center study, which may also affect the generalizability of the results, along with the retrospective nature of the investigation. This reflects the need for national quality registries with systemic and structured data entry to provide a coherent and cost‐effective way to monitor the quality and effectiveness of care and patient safety in the real‐world clinical setting.

## 5 CONCLUSION

In conclusion, targeted therapies, ibrutinib, idelalisib, and venetoclax, implicated benefits for patients with CLL over conventional chemo‐ or CIT regimens in both first‐ and second treatment lines, and the clinical outcomes mirror those reported in randomized clinical trials. The favorable OS times achieved by targeted therapies in both first and second lines suggest that the targeted agents should be used as early as possible, in order to improve treatment outcomes. The results also highlight the importance of routine assessment of del(17p), TP53 mutation and IGHV mutational status as these markers serve as a treatment guiding factor and affect the treatment outcomes.

## NOVELTY STATEMENT

What is the NEW aspect of your work? We here present the first retrospective registry study of real‐world Finnish CLL treatment patterns describing the outcomes of targeted therapy over more historical SOC treatments.

What is the CENTRAL finding of your work? Implementation of targeted therapies suggests improved treatment outcomes among the Finnish patients with CLL in first‐ and second‐line settings.

What is (or could be) the SPECIFIC clinical relevance of your work? Our findings suggest that previously reported benefits of targeted therapies can also be demonstrated in real‐life settings mandating their use in earlier lines of treatment.

## CONFLICT OF INTEREST

Juha Ranti served as a consultant for Janssen, AbbVie, and AstraZeneca, on the speakers' bureau for AbbVie and received travel support from Janssen and AbbVie. Katariina Perkonoja and Tommi Kauko are present or previous employees of Auria Clinical Informatics serving both academic research and industry‐sponsored scientific studies. Heidi Loponen and Emmi I. Joensuu are employees of MedEngine Oy. Tiina M. Järvinen is an employee of Janssen‐Cilag Oy.

## Supporting information

Table S1–S4

## References

[1] Finnish Cancer Registry . Cancer statistics. https://syoparekisteri.fi/tilastot/tautitilastot/ Last Accessed September 22, 2020.

[2] Kipps TJ , Stevenson FK , Wu CJ , Croce CM , Packham G , Wierda WG , et al. Chronic lymphocytic leukaemia. Nat Rev Dis Primers. 2017;3:16096. 28102226 10.1038/nrdp.2016.96 PMC5336551

[3] Hallek M , Shanafelt TD , Eichhorst B . Chronic lymphocytic leukaemia. Lancet 2018;391:1524–37. 29477250 10.1016/S0140-6736(18)30422-7

[4] Hallek M , Cheson BD , Catovsky D , Caligaris‐Cappio F , Dighiero G , Döhner H , et al. iwCLL guidelines for diagnosis, indications for treatment, response assessment, and supportive management of CLL. Blood 2018;131(25):2745–60. 29540348 10.1182/blood-2017-09-806398

[5] Keating MJ , O'Brien S , Albitar M , Lerner S , Plunkett W , Giles F , et al. Early results of a chemoimmunotherapy regimen of fludarabine, cyclophosphamide, and rituximab as initial therapy for chronic lymphocytic leukemia. J Clin Oncol. 2005;23(18):4079–88. 15767648 10.1200/JCO.2005.12.051

[6] Eichhorst B , Fink AM , Bahlo J , Busch R , Kovacs G , Maurer C , et al. First‐line chemoimmunotherapy with bendamustine and rituximab versus fludarabine, cyclophosphamide, and rituximab in patients with advanced chronic lymphocytic leukaemia (CLL10): an international, open‐label, randomised, phase 3, non‐inferiority trial. Lancet Oncol. 2016;17(7):928–42. 27216274 10.1016/S1470-2045(16)30051-1

[7] Goede V , Fischer K , Engelke A , Schlag R , Lepretre S , Montero LFC , et al. Obinutuzumab as frontline treatment of chronic lymphocytic leukemia: updated results of the CLL11 study. Leukemia 2015; 29(7):1602–4. 25634683 10.1038/leu.2015.14

[8] Eichhorst B , Robak T , Montserrat E , Ghia P , Hillmen P , Hallek M , et al. Chronic lymphocytic leukaemia: ESMO Clinical Practice Guidelines for diagnosis, treatment and follow‐up. Ann Oncol. 2015;26(Suppl 5):v78–84. 33091559 10.1016/j.annonc.2020.09.019

[9] Jain N , O'Brien S . The shifting paradigm in chronic lymphocytic leukemia: is chemotherapy still relevant? Cancer J. 2019;25(6):374–7. 31764117 10.1097/PPO.0000000000000417

[10] Moreno C , Greil R , Demirkan F , Tedeschi A , Anz B , Larratt L , et al. Ibrutinib plus obinutuzumab versus chlorambucil plus obinutuzumab in first‐line treatment of chronic lymphocytic leukaemia (iLLUMINATE): a multicentre, randomised, open‐label, phase 3 trial. Lancet Oncol. 2019;20(1):43–56. 30522969 10.1016/S1470-2045(18)30788-5

[11] Munir T , Brown JR , O'Brien S , Barrientos JC , Barr PM , Reddy NM , et al. Final analysis from RESONATE: up to six years of follow‐up on ibrutinib in patients with previously treated chronic lymphocytic leukemia or small lymphocytic lymphoma. Am J Hematol. 2019;94(12):1353–63. 31512258 10.1002/ajh.25638 PMC6899718

[12] Burger JA , Barr PM , Robak T , Owen C , Ghia P , Tedeschi A , et al. Long‐term efficacy and safety of first‐line ibrutinib treatment for patients with CLL/SLL: 5 years of follow‐up from the phase 3 RESONATE‐2 study. Leukemia 2020;34(3):787–98. 31628428 10.1038/s41375-019-0602-x PMC7214263

[13] Fraser GAM , Chanan‐Khan A , Demirkan F , Santucci Silva R , Grosicki S , Janssens A , et al. Final 5‐year findings from the phase 3 HELIOS study of ibrutinib plus bendamustine and rituximab in patients with relapsed/refractory chronic lymphocytic leukemia/small lymphocytic lymphoma. Leuk Lymphoma. 2020;61(13):3188–97. 32762271 10.1080/10428194.2020.1795159 PMC9094431

[14] Sharman JP , Coutre SE , Furman RR , Cheson BD , Pagel JM , Hillmen P , et al. Final results of a randomized, phase III study of rituximab with or without idelalisib followed by open‐label idelalisib in patients with relapsed chronic lymphocytic leukemia. J Clin Oncol. 2019;37(16):1391–402. 30995176 10.1200/JCO.18.01460 PMC10448866

[15] Al‐Sawaf O , Zhang C , Tandon M , Sinha A , Fink AM , Robrecht S , et al. Venetoclax plus obinutuzumab versus chlorambucil plus obinutuzumab for previously untreated chronic lymphocytic leukaemia (CLL14): follow‐up results from a multicentre, open‐label, randomised, phase 3 trial. Lancet Oncol. 2020;21(9):1188–200. 32888452 10.1016/S1470-2045(20)30443-5

[16] Kater AP , Wu JQ , Kipps T , Eichhorst B , Hillmen P , D'Rozario J , et al. Venetoclax plus rituximab in relapsed chronic lymphocytic leukemia: 4‐year results and evaluation of impact of genomic complexity and gene mutations from the MURANO phase III study. J Clin Oncol. 2020;38(34):4042–54. 32986498 10.1200/JCO.20.00948 PMC7768340

[17] Woyach JA , Ruppert AS , Heerema NA , Zhao W , Booth AM , Ding W , et al. Ibrutinib regimens versus chemoimmunotherapy in older patients with untreated CLL. N Engl J Med. 2018;379(26):2517–28. 30501481 10.1056/NEJMoa1812836 PMC6325637

[18] Shanafelt TD , Wang XV , Kay NE , Hanson CA , O'Brien S , Barrientos J , et al. Ibrutinib–rituximab or chemoimmunotherapy for chronic lymphocytic leukemia. N Engl J Med. 2019;381(5):432–43. 31365801 10.1056/NEJMoa1817073 PMC6908306

[19] Byrd JC , Gribben JG , Peterson BL , Grever MR , Lozanski G , Lucas DM , et al. Select high‐risk genetic features predict earlier progression following chemoimmunotherapy with fludarabine and rituximab in chronic lymphocytic leukemia: justification for risk‐adapted therapy. J Clin Oncol. 2006;24(3):437–43. 16344317 10.1200/JCO.2005.03.1021

[20] Malcikova J , Tausch E , Rossi D , Sutton LA , Soussi T , Zenz T , et al. ERIC recommendations for TP53 mutation analysis in chronic lymphocytic leukemia – update on methodological approaches and results interpretation. Leukemia 2018;32(5):1070–80. 29467486 10.1038/s41375-017-0007-7 PMC5940638

[21] Eichhorst B , Robak T , Montserrat E , Ghia P , Niemann CU , Kater AP , et al. Chronic lymphocytic leukaemia: ESMO clinical practice guidelines for diagnosis, treatment and follow‐up. Ann Oncol. 2021;32(1):23–33. 33091559 10.1016/j.annonc.2020.09.019

[22] Eketorp Sylvan S , Asklid A , Johansson H , Klintman J , Bjellvi J , Tolvgård S , et al. First‐line therapy in chronic lymphocytic leukemia: a Swedish nation‐wide real‐world study on 1053 consecutive patients treated between 2007 and 2013. Haematologica 2019;104(4):797–804. 30467205 10.3324/haematol.2018.200204 PMC6442960

[23] Asklid A , Winqvist M , Eketorp Sylvan S , Mattsson A , Björgvinsson E , Søltoft F , et al. Outcomes of second‐line treatment in chronic lymphocytic leukemia – a population‐based study from a well defined geographical region between 2003 and 2013. Leuk Lymphoma. 2017;58(5):1219–23. 27894205 10.1080/10428194.2016.1246727

[24] Curovic Rotbain E , Frederiksen H , Hjalgrim H , Rostgaard K , Egholm GJ , Zahedi B , et al. IGHV mutational status and outcome for patients with chronic lymphocytic leukemia upon treatment: a Danish nationwide population‐based study. Haematologica 2020;105(6):1621–9. 31582540 10.3324/haematol.2019.220194 PMC7271602

[25] Schemper M , Wakounig S , Heinze G . The estimation of average hazard ratios by weighted Cox regression. Stat Med. 2009;28(19):2473–89. 19472308 10.1002/sim.3623

[26] R Core Team. R: A language and environment for statistical computing. R Foundation for Statistical Computing, Vienna, Austria. https://www.R‐project.org/. 2020.

[27] Kater AP , Seymour JF , Hillmen P , Eichhorst B , Langerak AW , Owen C , et al. Fixed duration of venetoclax‐rituximab in relapsed/refractory chronic lymphocytic leukemia eradicates minimal residual disease and prolongs survival: post‐treatment follow‐up of the MURANO phase III study. J Clin Oncol. 2019;37(4):269–77. 30523712 10.1200/JCO.18.01580

[28] Hillmen P , Rawstron AC , Brock K , Muñoz‐Vicente S , Yates FJ , Bishop R , et al. Ibrutinib plus venetoclax in relapsed/refractory chronic lymphocytic leukemia: the CLARITY study. J Clin Oncol. 2019;37(30):2722–9. 31295041 10.1200/JCO.19.00894 PMC6879312

[29] Finnish Hematology Association (SHY) . Finnish CLL treatment guidelines. https://www.hematology.fi/fi/hoito‐ohjeet/veritaudit/kll/hoito Last Accessed February 4, 2021.

[30] Lindström V , Hakkarainen KM , Mehtälä J , Klement R , Leval A , Järvinen TM . Observational evidence from patients diagnosed with chronic lymphocytic leukaemia (CLL) in Finland between 2005–2015 show improved survival over time. Eur J Haematol. 2019;103(3):190–9. 31210368 10.1111/ejh.13273 PMC6851967

[31] Fischer K , Bahlo J , Fink AM , Goede V , Herling CD , Cramer P , et al. Long‐term remissions after FCR chemoimmunotherapy in previously untreated patients with CLL: Updated results of the CLL8 trial. Blood 2016;127(2):208–15. 26486789 10.1182/blood-2015-06-651125

[32] Kutsch N , Bahlo J , Robrecht S , Franklin J , Zhang C , Maurer C , et al. Long term follow‐up data and health‐related quality of life in frontline therapy of fit patients treated with FCR versus BR (CLL10 trial of the GCLLSG). Hemasphere 2020;4(1):e336. 32072150 10.1097/HS9.0000000000000336 PMC7000471

[33] Coutré SE , Barrientos JC , Brown JR , De Vos S , Furman RR , Keating MJ , et al. Management of adverse events associated with idelalisib treatment: expert panel opinion. Leuk Lymphoma. 2015;56(10):2779–86. 25726955 10.3109/10428194.2015.1022770 PMC4732460

[34] Zenz T , Mertens D , Küppers R , Döhner H , Stilgenbauer S . From pathogenesis to treatment of chronic lymphocytic leukaemia. Nat Rev Cancer. 2010;10(1):37–50. 19956173 10.1038/nrc2764

[35] Thompson PA , Tam CS , O'Brien SM , Wierda WG , Stingo F , Plunkett W , et al. Fludarabine, cyclophosphamide, and rituximab treatment achieves long‐term disease‐free survival in IGHV‐mutated chronic lymphocytic leukemia. Blood 2016;127(3):303–9. 26492934 10.1182/blood-2015-09-667675 PMC4760129

[36] Mato AR , Barrientos JC , Ghosh N , Pagel JM , Brander DM , Gutierrez M , et al. Prognostic testing and treatment patterns in chronic lymphocytic leukemia in the era of novel targeted therapies: results from the informCLL Registry. Clin Lymphoma Myeloma Leuk. 2020;20(3):174‐183.e3. 32033927 10.1016/j.clml.2019.10.009 PMC7890939

[37] Charlson ME , Pompei P , Ales KL , MacKenzie CR . A new method of classifying prognostic comorbidity in longitudinal studies: development and validation. J Chronic Dis. 1987;40(5):373–83. 3558716 10.1016/0021-9681(87)90171-8

