# Supplementary material for: Characterization of real‐world treatment practices and outcomes among patients with chronic lymphocytic leukemia treated in a Finnish tertiary center
Source: EJHaem. 2021 Nov 21;3(1):291–300. doi: 10.1002/jha2.322 (PMC9176063; doi:10.1002/jha2.322)
Supplement: Supplementary file 1 — Table S1–S4 [file JHA2-3-291-s001.docx]

**Supplementary Table 1.** Median follow-up times (Q1, Q3) in months in the studied subgroups.

|  | **Treatment line 1** | **Treatment line 2** |
| --- | --- | --- |
| **Treatment groups** | | |
| Targeted | 16.0 (13.7, 21.0), N=8 | 11.2 (9.8, 19.4), N=13 |
| SOC | 36.8 (10.8, 71.0), N=114 | 30.2 (9.0, 56.7), N=47 |
| **Treatment periods** | | |
| Early (2005–2013) | 57.1 (25.5, 82.7), N=74 | 27.6 (11.5, 67.3), N=27 |
| Late (2014–2019) | 18.0 (6.6, 31.6), N=48 | 17.6 (7.9, 39.6), N=33 |
| **IGHV mutational status** | | |
| Unmutated | 37.3 (17.3, 63.9), N=49 | 19.4 (8.7, 38.0), N=35 |
| Mutated | 35.2 (11.6, 84.9), N=25 | 33.0 (9.1, 55.4), N=11 |
| Unknown | 27.7 (9.7, 61.4), N=48 | 36.8 (16.2, 63.1), N=14 |
| **TP53 aberration** | | |
| Yes | 46.1 (23.3, 70.5), N=16 | 26.7 (10.6, 38.0), N=12 |
| No | 26.6 (11.1, 51.7), N=34 | 14.9 (7.6, 42.4), N=19 |
| Unknown | 37.4 (10.2, 71.5), N=72 | 33.0 (9.5, 49.4), N=29 |
| **High-risk** | | |
| Yes | 36.5 (16.6, 63.8), N=54 | 19.0 (7.9, 37.1), N=36 |
| No | 36.6 (24.1, 63.4), N=8 | 17.6 (10.2, 45.1), N=5 |
| Unknown | 35.1 (9.3, 71.5), N=60 | 36.9 (18.9, 71.0), N=19 |

Targeted, targeted therapy (ibrutinib, idelalisib, or venetoclax as monotherapy or in combination); SOC, standard of care therapy (any other therapy excluding targeted therapies); TP53 aberration, TP53 mutation and/or 17p deletion; high-risk, 17p deletion, 11q deletion, TP53 mutation or unmutated IGHV; yes, TP53 aberration or high-risk markers detected; no, no TP53 aberration or high-risk markers detected; unknown, IGHV mutational status, TP53 aberration, or risk status not tested.

**Supplementary Table 2.** Treatment sequencing from first to second and second to third treatment line.

| **Treatment group (line 1)** | Antibody-based | B/BR | Chlorambucil-based | FC/FCR | Other | Targeted | Overall |
| --- | --- | --- | --- | --- | --- | --- | --- |
|  | (N=20) | (N=23) | (N=41) | (N=25) | (N=5) | (N=8) | (N=122) |
| **Treatment group (line 2)** | |  |  |  |  |  |  |
| Antibody-based | 7 (35.0%) | 0 (0%) | 2 (4.9%) | 4 (16.0%) | 0 (0%) | 0 (0%) | 13 (10.7%) |
| B/BR | 1 (5.0%) | 2 (8.7%) | 1 (2.4%) | 2 (8.0%) | 1 (20.0%) | 1 (12.5%) | 8 (6.6%) |
| Chlorambucil-based | 0 (0%) | 1 (4.3%) | 11 (26.8%) | 1 (4.0%) | 0 (0%) | 0 (0%) | 13 (10.7%) |
| FC/FCR | 1 (5.0%) | 0 (0%) | 1 (2.4%) | 6 (24.0%) | 2 (40.0%) | 0 (0%) | 10 (8.2%) |
| Other | 0 (0%) | 0 (0%) | 2 (4.9%) | 1 (4.0%) | 0 (0%) | 0 (0%) | 3 (2.5%) |
| Targeted | 1 (5.0%) | 7 (30.4%) | 1 (2.4%) | 2 (8.0%) | 0 (0%) | 2 (25.0%) | 13 (10.7%) |
| No second-line treatment | 10 (50.0%) | 13 (56.5%) | 23 (56.1%) | 9 (36.0%) | 2 (40.0%) | 5 (62.5%) | 62 (50.8%) |
|  | | | | | | | |
| **Treatment group (line 2)** | Antibody-based | B/BR | Chlorambucil-based | FC/FCR | Other | Targeted | Overall |
|  | (N=13) | (N=8) | (N=13) | (N=10) | (N=3) | (N=13) | (N=60) |
| **Treatment group (line 3)** | |  |  |  |  |  |  |
| Antibody-based | 3 (23.1%) | 0 (0%) | 0 (0%) | 1 (10.0%) | 1 (33.3%) | 1 (7.7%) | 6 (10.0%) |
| B/BR | 0 (0%) | 0 (0%) | 0 (0%) | 1 (10.0%) | 0 (0%) | 0 (0%) | 1 (1.7%) |
| Chlorambucil-based | 0 (0%) | 0 (0%) | 3 (23.1%) | 0 (0%) | 0 (0%) | 0 (0%) | 3 (5.0%) |
| FC/FCR | 0 (0%) | 0 (0%) | 0 (0%) | 2 (20.0%) | 1 (33.3%) | 0 (0%) | 3 (5.0%) |
| Other | 2 (15.4%) | 0 (0%) | 1 (7.7%) | 0 (0%) | 0 (0%) | 0 (0%) | 3 (5.0%) |
| Targeted | 2 (15.4%) | 4 (50.0%) | 1 (7.7%) | 2 (20.0%) | 0 (0%) | 2 (15.4%) | 11 (18.3%) |
| No third-line treatment | 6 (46.2%) | 4 (50.0%) | 8 (61.5%) | 4 (40.0%) | 1 (33.3%) | 10 (76.9%) | 33 (55.0%) |

Antibody-based, monoclonal antibody-based therapy (monotherapy or in combination; other than FCR or BR); B, bendamustine; BR, bendamustine-rituximab; chlorambucil-based, chlorambucil-based therapy (monotherapy or in combination with obinutuzumab, ofatumumab, or rituximab); FC, fludarabine- cyclophosphamide; FCR, fludarabine-cyclophosphamide-rituximab; other, other therapy excluding regimens in any other category; targeted, targeted therapy (ibrutinib, idelalisib, or venetoclax as monotherapy or in combination).

**Supplementary Table 3.** Multivariate analysis results.

|  | **Treatment line 1** | | **Treatment line 2** | |
| --- | --- | --- | --- | --- |
|  | Hazard ratio (95% CI) | P-value | Hazard ratio (95% CI) | P-value |
| **OS** | | | | |
| Female sex (vs. male) | 1.39 (0.63–3.10) | 0.414 | 0.33 (0.08–1.39) | 0.130 |
| Targeted treatment (vs. SOC) | NE^†^ | NE^†^ | 0.60 (0.13–2.71) | 0.504 |
| Treatment initiation year (per year) | 1.18 (1.02–1.37) | **0.023*** | 1.04 (0.83–1.30) | 0.754 |
| With TP53 aberration (vs. without) | 2.63 (0.58–12.02) | 0.211 | 1.93 (0.22–16.71) | 0.549 |
| TP53 aberration status unknown (vs. without) | 10.24 (3.22–32.57) | **<0.001***** | 7.69 (1.81–32.56) | **0.006**** |
| IGHV unmutated (vs. IGHV mutated) | 1.57 (0.66–3.73) | 0.308 | 2.82 (0.27–29.10) | 0.383 |
| Age at baseline (per year) | 1.06 (1.01–1.12) | **0.026*** | 1.06 (0.98–1.14) | 0.166 |
| Binet C (vs. other) | 0.45 (0.22–0.92) | **0.030*** | 0.28 (0.10–0.81) | **0.019*** |
| Comorbidity class^a^ 1–2 (vs. 0) | 0.59 (0.19–1.86) | 0.365 | 0.27 (0.05–1.40) | 0.119 |
| Comorbidity class^a^ ≥3 (vs. 0) | 0.78 (0.24–2.60) | 0.690 | 0.50 (0.08–2.97) | 0.445 |
| **TTNT** | | | | |
| Female sex (vs. male) | 1.67 (0.67–4.17) | 0.273 | 0.32 (0.03–3.56) | 0.356 |
| Targeted treatment (vs. SOC) | 0.96 (0.19–4.94) | 0.959 | 0.21 (0.07–0.62) | **0.005**** |
| Treatment initiation year (per year) | 1.20 (1.05–1.36) | **0.007**** | 0.97 (0.69–1.35) | 0.835 |
| With TP53 aberration (vs. without) | 1.25 (0.59–2.67) | 0.562 | 1.39 (0.27–7.17) | 0.693 |
| TP53 aberration status unknown (vs. without) | 0.86 (0.35–2.09) | 0.737 | 1.16 (0.26–5.29) | 0.845 |
| IGHV unmutated (vs. IGHV mutated) | 3.43 (1.46–8.02) | **0.005**** | 4.70 (1.16–19.01) | **0.030*** |
| Age at baseline (per year) | 1.01 (0.98–1.05) | 0.482 | 1.05 (0.97–1.13) | 0.208 |
| Binet C (vs. other) | 1.46 (0.80–2.65) | 0.216 | 0.22 (0.07–0.63) | **0.005**** |
| Comorbidity class^a^ 1–2 (vs. 0) | 1.29 (0.51–3.30) | 0.589 | 0.35 (0.08–1.58) | 0.171 |
| Comorbidity class^a^ ≥3 (vs. 0) | 0.97 (0.42–2.26) | 0.949 | 0.96 (0.22–4.28) | 0.958 |

^†^NE, not evaluable due to the small number of cases in the targeted group. Statistically significant **P*<0.05, ***P*<0.01, ****P*<0.001; OS, overall survival; TTNT, time-to-next-treatment; TP53 aberration, TP53 mutation and/or 17p deletion; CI, confidence interval; targeted, targeted therapy (ibrutinib, idelalisib, or venetoclax as monotherapy or in combination); SOC, standard of care therapy (any other therapy excluding targeted therapies). ^a^The comorbidity index was defined according to the Charlson comorbidity index (37) with the range of 0–10 in the dataset.

**Supplementary Table 4.** Treatment outcomes (OS, TTNT) in patients with high-risk disease features

| **IGHV mutational status** | | | | | | | | |
| --- | --- | --- | --- | --- | --- | --- | --- | --- |
|  | **Treatment line 1** | | | | **Treatment line 2** | | | |
|  | **Unmutated (N=49)** | **Mutated (N=25)** | **Unknown (N=48)** | **P-value^†^** | **Unmutated (N=35)** | **Mutated (N=11)** | **Unknown (N=14)** | **P-value^†^** |
| mOS, (95% CI) | 63.9 (55.9–NR) | 92.1 (35.2–NR) | 36.0 (16.5–59.1) | **0.005**** | 33.3 (26.5–NR) | 100.5 (33.0–NR) | 36.8 (24.2–NR) | 0.388 |
| mTTNT, (95% CI) | 33.0 (19.2–44.3) | 68.1 (37.6–NR) | 59.1 (30.2–NR) | **0.001**** | 23.7 (14.0–NR) | NR (48.3–NR) | 41.3 (30.8–NR) | **0.018*** |
| Deaths, % (n) | 44.9 (22) | 48.0 (12) | 79.2 (38) |  | 45.7 (16) | 45.5 (5) | 85.7 (12) |  |
| **TP53 aberration** | | | | | | | | |
|  | **Treatment line 1** | | | | **Treatment line 2** | | | |
|  | **No (N=34)** | **Yes (N=16)** | **Unknown (N=72)** | **P-value^†^** | **No (N=19)** | **Yes (N=12)** | **Unknown (N=29)** | **P-value^†^** |
| mOS, (95% CI) | NR (58.3–NR) | 72.7 (63.9–NR) | 38.6 (35.0–61.1) | **0.004**** | NR (NR–NR) | 66.9 (26.5–NR) | 33.0 (24.2–49.4) | 0.177 |
| mTTNT, (95% CI) | 28.4 (24.3–NR) | 35.1 (15.2–NR) | 54.1 (37.6–83.6) | **0.005**** | 30.5 (11.1–NR) | 23.7 (12.8–NR) | 41.3 (26.2–NR) | 0.217 |
| Deaths, % (n) | 20.6 (7) | 37.5 (6) | 81.9 (59) |  | 21.1 (4) | 41.7 (5) | 82.8 (24) |  |
| **High-risk** | | | | | | | | |
|  | **Treatment line 1** | | | | **Treatment line 2** | | | |
|  | **No (N=8)** | **Yes (N=54)** | **Unknown (N=60)** | **P-value^†^** | **No (N=5)** | **Yes (N=36)** | **Unknown (N=19)** | **P-value^†^** |
| mOS, (95% CI) | NR (NR–NR) | 63.9 (55.9–NR) | 37.0 (26.3–68.1) | **0.013*** | NR (NR–NR) | 33.3 (26.5–NR) | 36.9 (27.6–NR) | NA |
| mTTNT, (95% CI) | 28.4 (27.0–NR) | 33.3 (25.5–47.1) | 59.5 (39.6–NR) | **0.003**** | 48.3 (NR–NR) | 23.7 (14.0–NR) | 70.2 (30.8–NR) | NA |
| Deaths, % (n) | 12.5 (1) | 44.4 (24) | 78.3 (47) |  | 20 (1) | 44.4 (16) | 84.2 (16) |  |

^†^P-value, log-rank p-value; Statistically significant **P*<0.05, ***P*<0.01; mOS, median overall survival; mTTNT, median time-to-next-treatment; TP53 aberration, TP53 mutation and/or 17p deletion; high-risk, 17p deletion, 11q deletion, TP53 mutation or unmutated IGHV; no, no TP53 aberration or high-risk markers detected; yes, TP53 aberration or high-risk markers detected; unknown, IGHV mutational status, TP53 aberration, or risk status not tested; NR, not reached; NA, not applicable (statistical analyses not conducted due to the small sample size in the non-high-risk group).
